# Mechanisms of Naturally Acquired Immunity to *Streptococcus pneumoniae*

**DOI:** 10.3389/fimmu.2019.00358

**Published:** 2019-03-01

**Authors:** Elisa Ramos-Sevillano, Giuseppe Ercoli, Jeremy S. Brown

**Affiliations:** Centre for Inflammation and Tissue Repair, UCL Respiratory, London, United Kingdom

**Keywords:** immunity, pneumococcus (*Streptococcus pneumoniae*), natural acquired immunity, pneumonia, protection

## Abstract

In this review we give an update on the mechanisms of naturally acquired immunity against *Streptococcus pneumoniae*, one of the major human bacterial pathogens that is a common cause of pneumonia, septicaemia, and meningitis. A clear understanding of the natural mechanisms of immunity to *S. pneumoniae* is necessary to help define why the very young and elderly are at high risk of disease, and for devising new prevention strategies. Recent data has shown that nasopharynx colonization by *S. pneumoniae* induces antibody responses to protein and capsular antigens in both mice and humans, and also induces Th17 CD4+ cellular immune responses in mice and increases pre-existing responses in humans. These responses are protective, demonstrating that colonization is an immunizing event. We discuss the data from animal models and humans on the relative importance of naturally acquired antibody and Th17 cells on immunity to *S. pneumoniae* at three different anatomical sites of infection, the nasopharynx (the site of natural asymptomatic carriage), the lung (site of pneumonia), and the blood (site of sepsis). Mouse data suggest that CD4+ Th17 cells prevent both primary and secondary nasopharyngeal carriage with no role for antibody induced by previous colonization. In contrast, antibody is necessary for prevention of sepsis but CD4+ cellular responses are not. Protection against pneumonia requires a combination of both antibody and Th17 cells, in both cases targeting protein rather than capsular antigen. Proof of which immune component prevents human infection is less easily available, but two recent papers demonstrate that human IgG targeting *S. pneumoniae* protein antigens is highly protective against septicaemia. The role of CD4+ responses to prior nasopharyngeal colonization for protective immunity in humans is unclear. The evidence that there is significant naturally-acquired immunity to *S. pneumoniae* independent of anti-capsular polysaccharide has clinical implications for the detection of subjects at risk of *S. pneumoniae* infections, and the data showing the importance of protein antigens as targets for antibody and Th17 mediated immunity should aid the development of new vaccine strategies.

## Introduction

*Streptococcus pneumoniae* is a major cause of acute otitis media, community-acquired pneumonia, bacterial sepsis, and meningitis and is estimated to be responsible for over 800,000 deaths annually in children (1). *S. pneumoniae* strains causing invasive infections are surrounded by a polysaccharide capsule layer that inhibits innate and adaptive immune responses to infection (2). The capsule has a varied biochemical composition and antigenic structure between *S. pneumoniae* strains, resulting in over 90 different capsular serotypes (3). Serotype specific *S. pneumoniae* vaccines have been developed using capsular antigen linked to carrier protein, termed pneumococcal conjugated vaccines (PCVs), and these are highly effective against the serotypes included in the vaccine (4–6). However, there is still a high level of mortality and morbidity due to *S. pneumoniae* infections due to the restricted serotype coverage of PCVs resulting in serotype-replacement disease, and their high cost leading to incomplete use of PCVs in low and middle-income countries. In addition, PCVs are less effective at preventing pneumonia compared to septicaemia and meningitis, despite pneumonia making the largest contribution to the burden of disease (1, 7, 8). Novel approaches are needed to overcome the limitations of the present PCV. One way of identifying new preventative strategies could be to define the mechanisms of natural adaptive immunity to *S. pneumoniae*, which could then be targeted to enhance immunity against infection in high-risk groups such as children and older adults. In this review we will discuss the evidence for naturally acquired adaptive immunity to *S. pneumoniae* and the mechanisms by which this maybe mediated.

## Clinical and Epidemiological Evidence for Adaptive Immunity to *S. pneumoniae*

### *S. pneumoniae* Nasopharyngeal Colonization

Unlike some major bacterial respiratory pathogens such as *Mycobacterium tuberculosis, S. pneumoniae* is also a ubiquitous human commensal of the upper respiratory tract, specifically of the nasopharynx. Colonization of the nasopharynx occurs in the first years of life and repeatedly thereafter (9–11). Successive episodes of colonization with different pneumococcal strains occur in children up to 2 years of age (9, 10), with the point prevalence of nasopharyngeal carriage estimated at 27–65% among infants. This colonization prevalence decreases with age to < 10% during adult life (12–14), and even lower rates in the elderly (15). This decline in colonization seems to have a multifactorial nature, one element of could be maturation of the immune system with age. The main nasopharyngeal reservoir for the spread of *S. pneumoniae* is in children, and consequently vaccination of children with PCV (which prevents nasopharyngeal colonization) has resulted in significant herd immunity against adult *S. pneumoniae* pneumonia (12).

### Epidemiological Evidence for Naturally Acquired Adaptive Immunity to *S. pneumoniae*

Subjects with defects of their adaptive immune response are more susceptible to *S. pneumoniae* infections. These groups include people with genetic or acquired defects in immunoglobulin production, and those who have severe defects of adaptive immunity due to stem cell transplantation or HIV infection (16–18). In addition, subjects with deficiencies in the classical pathway of complement activation have a massively increased incidence of pneumococcal septicaemia, meningitis, and bacterial pneumonia (19). Although this will reflect weakened complement-mediated innate immunity to *S. pneumoniae* (20), the classical complement pathway is also vital for antibody mediated killing of *S. pneumoniae* (2), and hence the susceptibility of subjects with classical pathway defects will partially reflect weakened antibody mediated immunity. Furthermore, the incidence of *S. pneumoniae* infection with age has a pronounced U shaped curve with the highest incidence in infants and the elderly (21), a pattern that suggests there is an adaptive immune response to *S. pneumoniae* that improves in children with maturity then perhaps wanes due to immunosenescence in the elderly. The frequency of *S. pneumoniae* septicaemia associated with pneumonia is also higher in infants than adults, indicating that in adults the immune system is able to prevent spread to the blood from the lungs. Overall, these epidemiological data indicate that there is significant naturally acquired adaptive immunity that helps prevent *S. pneumoniae* infections. Importantly, the incidence of infection with all serotypes falls at the same time (22), an epidemiological observation that suggests naturally acquired resistance to *S. pneumoniae* infections is mediated by serotype independent mechanisms rather than anticapsular antibody.

This evidence of naturally acquired adaptive immunity to *S. pneumoniae* leads to the question of how this has developed? Only a small proportion of humans have been exposed to *S. pneumoniae* during invasive diseases such as septicaemia and meningitis, and although many more humans will have had an episode of *S. pneumoniae* pneumonia this would have been with a single strain and capsular serotype. In contrast, almost all humans have been exposed to *S. pneumoniae* via nasopharyngeal colonization events on multiple occasions and by many different strains. Hence, if most (if not all) humans have a naturally acquired serotype-independent adaptive immunity to *S. pneumoniae* then this must have occurred because of colonization rather than active infectious disease.

### Evidence That *S. pneumoniae* Nasopharyngeal Colonization Is an Immunizing Event

Data obtained during natural and experimental human infection and from mouse models have now shown conclusively that colonization of the nasopharynx by *S. pneumoniae* is indeed an immunizing event. Multiple longitudinal studies of serum antibody responses in infants show the development of antibody responses to *S. pneumoniae* capsular and protein antigens in serum and saliva after human colonization events (11, 14, 23, 24). In murine models, several authors have shown both antibody and T-cell mediated immune responses develop after a colonization event with *S. pneumoniae* or exposure to *S. pneumoniae* antigens in the nasopharynx (25–32). In addition, after successful experimental human colonization with *S. pneumoniae* pre-existing antibody responses (33, 34) and *S. pneumoniae*-specific blood and lung IL-17 producing CD4^+^ memory cells are increased (35), providing enhanced humoral and cellular immunity. These data confirm that colonization is an immunizing event, and this is supported by data from infants showing gradual increases in anti-protein antigen IgG responses with age and correlations between colonization events and increases in anti-capsular antibody for several serotypes (11).

### Mechanisms of Naturally Acquired Adaptive Immune Protection Against *S. pneumoniae* Infection

The data discussed above demonstrate that humans do develop naturally acquired adaptive immune responses against *S. pneumoniae* in response to nasopharyngeal colonization and that these responses are likely to be protective. The adaptive response to nasopharyngeal colonization by *S. pneumoniae* includes acquisition of anti-capsular (14, 36, 37) and anti-protein antibodies (38–40), as well as CD4^+^ cellular immune responses targeting protein antigens alone (39, 41). The observation of CD4^+^ cellular immune responses is important, as Th17 CD4^+^ responses are a common mechanism for pathogen clearance at mucosal surfaces via rapid recruitment of neutrophils to the site of infection, improved epithelial barrier function, and increased secretion of antibacterial peptides (42, 43). However, to effectively utilize these findings to design preventative strategies against *S. pneumoniae* it is necessary to go one step further and identify which of these responses actually protect against *S. pneumoniae* infections.

All currently licensed pneumococcal vaccines use polysaccharide capsular antigen, and capsular antibodies against the capsule are highly protective against infection caused by that serotype (5, 44, 45). As a consequence, historically the literature has also emphasized the role of anti-capsule antibodies as the mechanism for naturally acquired immunity against *S. pneumoniae* (46). This is supported by the early data showing that sera from survivors of severe *S. pneumoniae* provided serotype-specific protection when used as a passive antibody treatment (47–51). However, as mentioned above the epidemiological evidence suggests that naturally acquired immunity has a significant serotype-independent component. Analysis of datasets from several countries (e.g., USA, Finland and Israel), demonstrated a decrease in incidence of invasive disease with age independent of increases in serotype-specific antibody levels, suggesting a different mechanism of protection such as acquired immunity to non-capsular antigens or maturation of non-specific immune responses (22). Theoretical modeling also suggested that only 30–60% of the reduction in recolonization is led by anti-capsular immunity generated during previous colonization events (52). Animal model data has shown that antibody and more recently cell mediated immune responses targeting protein rather than capsular antigens can protect against *S. pneumoniae* infections, providing proof of principle that these capsular-antigen independent mechanisms could mediate naturally acquired immunity to *S. pneumoniae* in humans (25, 26, 38, 53). Which proteins dominant the human antibody response to *S. pneumoniae* protein antigens was recently defined by Croucher using a proteome microarray that displays more than 2,000 potential *S. pneumoniae* antigens. Of these, 208 had significant IgG responses in sera obtained from 35 healthy US adults. About half of these antigens were allelic variants of the highly variable surface proteins PspA, PspC, ZmpA, and ZmpB expressed by different *S. pneumoniae* strains (54). The remaining proteins recognized by human sera were more conserved between strains and were enriched in motifs for adhesion or degradation, cell wall metabolism, or solute binding for transport. These results are consistent with a previous screen reverse vaccinology approach that identified proteins recognized by antibody in human sera which identified the proteins PspC and PspA, along with PcsB and StkP (55). There are only limited data on the identification of *S. pneumoniae* antigens that induce Th17 CD4^+^ cell responses as this is technically difficult. The available data was obtained in mice by screening of an expression library containing >96% of predicted pneumococcal protein and identified several proteins (including ABC transporter lipoproteins) that induce Th17 responses after exposure to whole pneumococci and as purified protein antigens, with little overlap between with antibody-inducing protein antigens (56).

Overall, the data from human and animal studies show colonization induces both anti-capsule and anti-protein antibody responses, and in addition cell mediated immunity to protein antigens. The anatomy, associated immunological tissues, and interactions with soluble immune effectors in mucosal lining fluid or in the plasma all vary between the common anatomical sites of *S. pneumoniae* infection, creating potential variations in the relative efficacy of different immune effectors for controlling *S. pneumoniae* infection between anatomical sites. Hence the following discussion of which mechanisms of naturally acquired adaptive immunity protect against *S. pneumoniae* is divided into three representative sites of infection; (i) the nasopharynx as a mucosal site colonized by *S. pneumoniae*; (ii) the blood as a site of systemic invasive infection; and (iii) the lungs as a deep site of mucosal infection intimately associated with the systemic circulation, and also the site responsible for the biggest burden of severe infections globally. In general, data from animal studies (mainly in mice) can clearly define protective mechanisms but is of more limited utility due to potential differences in immune function between species. However, after early childhood all humans have had multiple episodes of *S. pneumoniae* colonization each of which will stimulate some degree of immune response, and this background situation of considerable prior exposure to *S. pneumoniae* over many years is difficult to replicate in mouse models. Human data is directly relevant but it is usually only possible to provide potential correlations rather than direct proof between a specific immune effector and protection against disease.

### Prevention of Subsequent Colonization

#### Mouse Data

Whether antibodies have an important role in controlling a primary episode of nasopharyngeal colonization by *S. pneumoniae* was investigated by McCool and Weiser using murine models of infection and mice knock out strains affecting specific aspects of antibody mediated immunity (38). They established a murine model of colonization in *xid* mice, which have a poor antibody response to polysaccharide capsule. *S. pneumoniae* clearance from the nasopharynx was similar in *xid* mice compared to wild-type mice. These results were corroborated using *uMT* mice, which lack B cell and antibody responses, which were also still able to clear *S. pneumoniae* nasopharyngeal colonization. These results demonstrate that antibody responses do not aid clearance of primary colonization events, and have been confirmed by other studies (57). Instead, protection against recolonization was lost in mice deficient in CD4^+^ cells, highlighting the requirement of cellular immunity rather than antibody in protection against recolonization (58). Similar data were obtained using administration of the *S. pneumoniae* antigen cell wall polysaccharide intranasally, which inhibited subsequent colonization independent of antibody but dependent on CD4^+^ cells (29). In addition, these authors confirmed for the first time an important role for IL-17 in preventing colonization, although the exact mechanism by which this cytokine provided protection was not determined. Vaccination with killed unencapsulated *S. pneumoniae* also induced Th17 CD4^+^ immunity which prevented nasopharyngeal colonization with a heterologous *S. pneumoniae* serotype (27, 59).

The data obtained from these studies indicated an important role of CD4^+^ cells rather than antibodies for prevention of a second episode of *S. pneumoniae* colonization, and this has been confirmed by more detailed investigation in mice. Zhang et al. demonstrated that the clearance of both the initial and second episode of *S. pneumoniae* colonization in adult mice was dependent on cellular responses rather than humoral immunity (58, 60). Clearance of the initial episode of colonization involved recruitment of monocytes/macrophages into the upper airway lumen via a TLR2-dependent mechanism which required an IL-17 secreting CD4^+^ cell population (57, 60). Prevention of a second episode of colonization also required IL-17 and CD4^+^ cells, and was mediated by rapid neutrophil recruitment to the nasopharynx. These findings are supported by other data showing CD4^+^ cells and IL-17 are required for protection against colonization and mediate the protective effect of immunization with whole cell vaccines against colonization[28, 29, 31].

Overall, the murine data demonstrate that Th17 responses to protein antigens are critical for controlling *S. pneumoniae* colonization after a previous episode of colonization; subsequent work has identified several protein antigens that are able to induce Th17 mediated immunity but whether these antigens are the same for different *S. pneumoniae* strains is not known (56). These data do not preclude a potential role for vaccine induced antibody for prevention of colonization after vaccination, and in mice vaccination with protein antigens elicits protection against *S. pneumoniae* colonization (61, 62) and passive immunization with anticapsular antibodies prevents both colonization and transmission between littermates (63, 64).

#### Human Data

As discussed above longitudinal studies have demonstrated that antibodies to both protein and capsule antigens develop after episodes of *S. pneumoniae* colonization. As a consequence adult sera contains antibodies to multiple capsular *S. pneumoniae* serotypes and one hundred or so protein antigens (11, 54). In addition, different studies have identified Th17 CD4^+^ responses to *S. pneumoniae* in adenoid tissue (65, 66), bronchoalveolar lavage (35), and blood (31, 35, 67). Evidence for which of these naturally acquired immune mechanisms can prevent colonization in humans is less readily obtained. The impressive reduction in *S. pneumoniae* vaccine serotype prevalence as nasopharyngeal commensals in populations vaccinated with PCV demonstrates that high levels of anti-capsular antibody do prevent colonization (5–7). Recent data obtained using the experimental human pneumococcal carriage model (EHPC) and murine models suggest anticapsular antibody inhibits the establishment of colonization by serotype-specific agglutination of *S. pneumoniae* (63, 68). Whether the lower levels of anti-capsular antibody induced by natural colonization rather than vaccination with a PCV is enough to prevent colonization is not clear.

The most important data on immune mechanisms that prevent colonization in humans has been obtained using the EHPC model. In this experimental human infection model, human volunteers are inoculated intranasally with a serotype 6B strain leading to successful nasopharyngeal colonization in approximately half of subjects (34). Previous colonization prevented recolonization with the same strain (34). In this model, all recruited subjects had detectable IgG levels to *S. pneumoniae* antigens prior to nasopharyngeal administration of *S. pneumoniae*, but anti-pneumococcal IgG levels did not correlate with whether colonization was successful or not (34). In contrast, a previous EHPC model suggested that antibodies to the *S. pneumoniae* surface protein PspA correlated with prevention of successful colonization, providing some evidence that anti-protein antibody may prevent colonization (33). Exposure to serotype 6B or 23F strains increased antibody responses to multiple *S. pneumoniae* proteins (e.g., PspA, PspC, PsaA, and PdB), with the highest increases in carriage positive subjects (33, 34, 69). Systemic anti-capsule antibodies were only detected in subjects that developed carriage after challenge (34). *S. pneumoniae* specific IL-17 secreting CD4^+^ cells were found in the lung prior to exposure to the 6B strain but were increased in bronchoalveolar fluid 17-fold and in blood 8-fold in volunteers in which colonization was successful (35, 69). Overall these data confirm that pre-existing antibody and local CD4^+^ cellular immunity to *S. pneumoniae* are significantly boosted by colonization events, and that this seems to prevent recolonization for at least a period of several months. However, the data are unable to define which of these immune mechanisms was important for the prevention of recolonization.

### Prevention of *S. pneumoniae* Septicaemia and Meningitis

#### Mouse Data

Mouse data also clearly demonstrates that colonization of the nasopharynx promotes protection against invasive disease, but in contrast to prevention of colonization this is dependent on antibody mediated immunity, not CD4^+^ cells. Cohen et al. showed that a previous episode of colonization with the *S. pneumoniae* D39 strain was highly protective against subsequent lethal invasive pneumonia, with the major effect being prevention of septicaemia (70). CD4^+^ depleted mice were still protected against septicaemia, whereas no protection was seen in antibody deficient mice, and passive transfer of serum from colonized mice into immune naïve mice also protected against *S. pneumoniae* septicaemia. Similarly, Bou Ghanem *et al* found that previous colonization protected against *S. pneumoniae* pneumonia with septicaemia caused by the TIGR4 strain, with the most profound effect seen on blood colony forming units (CFU) (71). Repeated episodes of nasopharynx colonization in antibody deficient mice did not induced any protection whereas protection persisted after CD4^+^ cell depletion, and the authors proposed long-lived antibody secreting CD138^+^ cells were responsible for the protective effect of prior colonization. These data demonstrating the important role of antibody are perhaps not surprising as protection against septicaemia in mice is dependent on soluble mediators such as complement and naturally and induced antibody working in concert with the reticuloendothelial system, especially the spleen (72–74), rather than CD4^+^ Th17 mediated mechanisms.

#### Human Data

It was commonly accepted for many years that the major mechanism of natural immunity against invasive pneumococcal disease (IPD) was anti-capsular antibodies generated by previous episodes of either colonization or infection (44, 48, 50). However, this assumption has been progressively threatened by epidemiological data as discussed above. More recently experimental data by Wilson et al. has demonstrated that antibody to protein rather than capsular antigen is a major mechanism of naturally acquired immunity to *S. pneumoniae* septicaemia and IPD (75). To do so the authors used Intravenous Immunoglobulin (IVIG) which is used to prevent infections in individuals with primary antibody deficiency (76, 77). IVIG contains the pooled IgG from the plasma of approximately a thousand donors who are unlikely to have been vaccinated against *S. pneumoniae* due to their lack of risk factors, and therefore contains the naturally acquired antibody repertoire for the population from which the donors are obtained. Antibodies to both *S. pneumoniae* capsule and multiple proteins were detected in IVIG. Immunoblots against lysates from different pneumococcal strains demonstrated a highly conserved pattern of protein targets recognized by IVIG, including major pneumococcal surface proteins such as choline binding proteins and lipoproteins. Importantly, opsonophagocytosis of *S. pneumoniae* was improved against unencapsulated bacteria but reduced when anticapsular antibody was depleted from the IVIG preparation. IVIG was highly protective against *S. pneumoniae* septicaemia in mouse models, even after depletion of anticapsular antibody. These data demonstrate there is a high level of naturally acquired antibody to *S. pneumoniae* protein antigens in adult sera that promotes bacterial clearance during sepsis. In addition, passive vaccination of mice with sera obtained from human EHPC subjects colonized with the serotype 6B strain conferred protection against the D39 serotype 2 strain in a pneumonia model in which lethality is driven by septicaemia (34). The data from these two studies provide direct proof that antiprotein antibody is an important mechanism of naturally acquired immunity to *S. pneumoniae* systemic infection.

### Prevention of *S. pneumoniae* Pneumonia

The nasopharynx represents a mucosal site of infection, and septicaemia a systemic site, whereas the commonest site of serious infection, the lungs, represents a combination of the two. In the early stages of pneumonia infection is limited to interactions with the mucosal surface, which are similar to those found in the nasopharynx. As the infection develops there is a breakdown in the epithelial/endothelial barrier function and cellular recruitment to the alveoli, recruiting systemic soluble and white cell-mediated immune mechanisms to the alveoli. Hence, both mucosal and systemic mechanisms of adaptive immunity could potentially have roles in protection against *S. pneumoniae* pneumonia.

#### Mouse Data

Several studies have examined the role of colonization-induced immunity for protection against pneumonia in murine models. In a murine model of asymptomatic carriage with the D39 strain the number of CFU recovered from lung was reduced compared to control mice after a lethal pneumonia invasive challenge suggesting that colonization does improve local lung immunity against *S. pneumoniae* pneumonia (26). The authors also showed increased levels of IL-17A and CD4^+^ cells in lungs of previously colonized mice, indicating a potential role for T cell mediated immunity for prevention of pneumonia. These data were developed by Wilson et al. using a non-invasive *S. pneumoniae* serotype 19F strain that only causes lung infection without sepsis to allow lung immunity to be assessed separate to protection against systemic infection (25). Prior colonization protected against subsequent pneumonia, with a marked reduction in lung CFU. Additional experiments demonstrated that B cells, CD4^+^ and IL-17 were each necessary for protection against *S. pneumoniae* pneumonia caused by the 19F strain. These results suggested that naturally acquired protective immunity to *S. pneumoniae* pneumonia required a combination of both humoral immunity and Th17 CD4^+^ cells. Protection also required neutrophils, presumably as the main mechanism of bacterial killing, and previously colonized mice seemed to have a more rapid influx of neutrophils into the lungs during *S. pneumoniae* pneumonia compatible with improved local Th17 immunity (58). There was no detectable anti-capsular IgG responses after colonization. This suggested the humoral component of protection against pneumonia required anti-protein antibodies instead, and in agreement with previous mouse and human colonization models (28, 31, 69) the authors detected antibody responses to the important surface protein antigens PsaA, PhtD, and PpmA.

Two additional papers have also demonstrated an important role for Th17 CD4^+^ cells induced by prior exposure to *S. pneumoniae* for protection against subsequent severe *S. pneumoniae* pneumonia (78, 79). In both these papers protection was seen against pneumonia caused by a different capsular serotype, demonstrating that it was dependent on recognition of protein antigens. Smith et al. used prior exposure to *S. pneumoniae* through self-limiting lung infection rather than colonization, and demonstrated that protection was specific to *S. pneumoniae* and required administration of live rather than dead bacteria, suggesting bacterial replication within the respiratory tract was necessary. Importantly, they identified a population of tissue resident memory T cells within the lungs that mediated the antigen specific Th17 immunity to *S. pneumoniae* and that was restricted to the lobe that had been previously exposed to *S. pneumoniae*. It remains to be seen whether the low quantities of bacteria that reach the lung due to microaspiration from “pure” nasopharyngeal colonization are also sufficient to induce this population of lung tissue resident memory T cells.

#### Human Data

The increased incidence of lung infection in patients with immunoglobulin deficiencies (16, 80), HIV infection (81), or inherited disorders affecting IL-17 pathways [e.g., hyperIgE syndrome (82)], do indicate roles for both antibody and Th17 CD4^+^ cells for protection against *S. pneumoniae* pneumonia in humans as well as mice. However, these data are confounded by the presence of additional immune effects for each condition, with even immunoglobulin deficiencies resulting in functional impairment of *S. pneumoniae*-specific CD4^+^ cells (83). As previously mentioned, data obtained using bronchoalveolar lavage fluid has confirmed there are pre-existing CD4^+^ cells present in the human lung that produce IL-17 (and often TNFalpha) when stimulated with *S. pneumoniae* (36). The authors demonstrated that IL-17 improved *S. pneumoniae* killing mediated by either macrophages or neutrophils (31), providing an additional mechanism by which IL-17 can assist immunity to extracellular bacteria. BALF also contains antibody to *S. pneumoniae* protein and capsular antigens (33, 34, 69). Hence the human data has identified local pulmonary Th17 CD4^+^ and antibody specific for *S. pneumoniae* that are boosted by nasopharyngeal colonization which could potentially assist lung immunity to pneumonia as has been described in mouse models. However, the additional step of identifying which of these immune responses is actually protective against *S. pneumoniae* lung infection in humans is not easily achieved.

### Implications of Data on Naturally Acquired Immunity to *S. pneumoniae*

**Figure 1:**
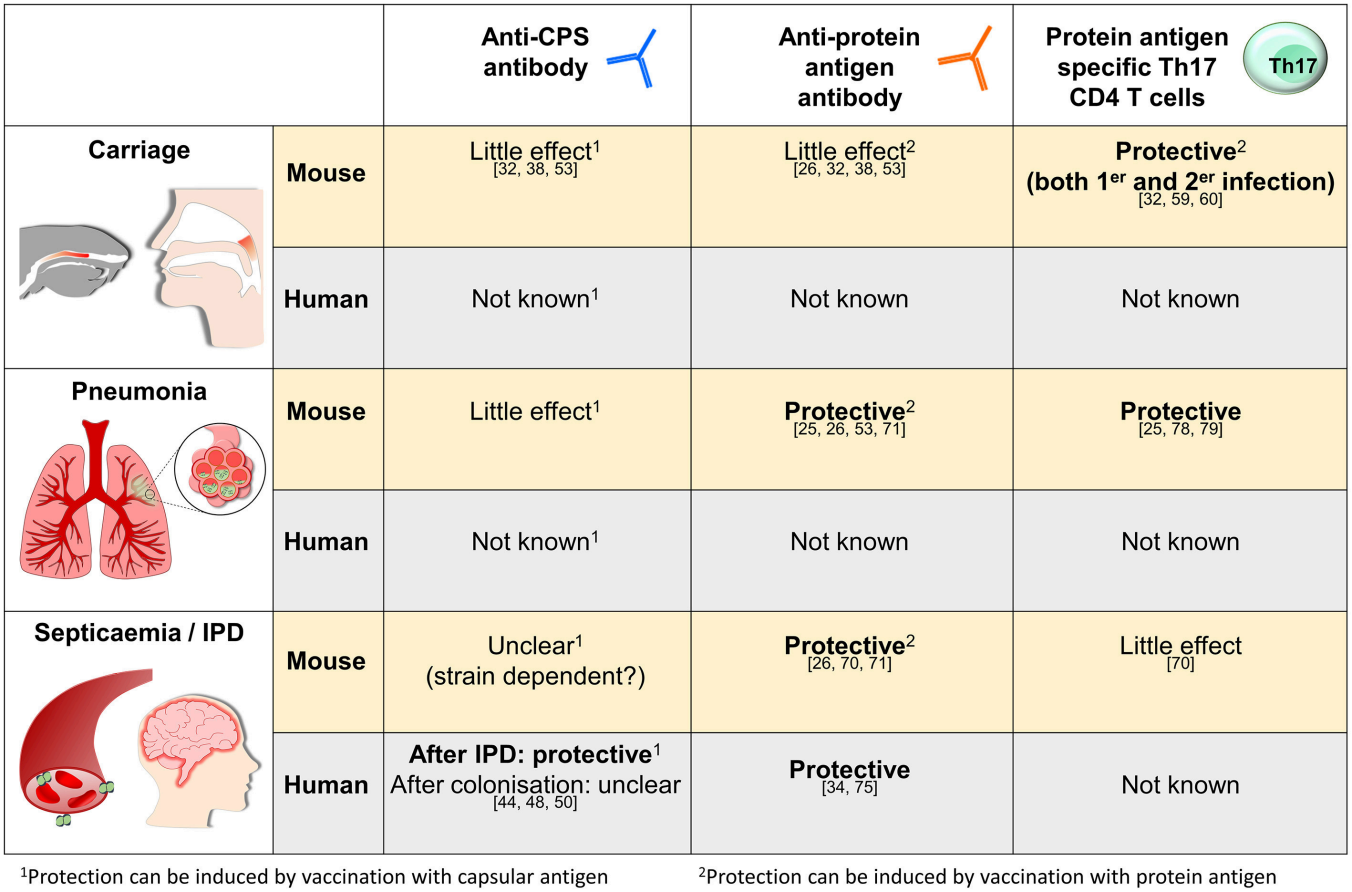
Summary of what is known about the mechanisms of naturally acquired (as opposed to vaccine-induced) adaptive immunity to *S. pneumoniae* in mice and humans for three representative sites of infection, the nasopharynx, the lungs, and in the blood. The references relevant for each statement are as shown.

As described above murine studies have defined how naturally acquired immunity due to prior colonization can protect against *S. pneumoniae* infections, and much of the data has been reinforced by epidemiological and experimental data obtained from humans. These mechanisms are summarized in Figure 1. In this last section, we will discuss how the data on naturally acquired infection may effect vaccination strategies and our understanding of the mechanisms underpinning susceptibility to *S. pneumoniae* in high risk groups.

In children, pneumococcal colonization events occur frequently and as discussed above this is necessary for the development of immunity against *S. pneumoniae*. During adulthood, colonization rates hugely decrease (15, 36, 84), but the data from the EHPC model clearly show that a colonization event still stimulates a boost to the pre-existing immune responses (34, 35, 69). Hence colonization has significant immune benefits to the host that could be affected if the ecology of *S. pneumoniae* colonization is altered. Multiple studies have indeed shown that routine PCV vaccination in children has profound effects on *S. pneumoniae* colonization, with hugely decreased carriage rates of the serotypes present in the vaccine (5, 6). However, there has been a parallel increase in colonization with non-vaccine serotypes reported to have a lower invasive disease potential (85, 86), and this alteration of carriage serotype ecology in favor or less invasive serotypes should also maintain the beneficial effects of colonization on natural mechanisms of immunity. If more aggressive preventative strategies reduce overall *S. pneumoniae* carriage rates then there might be a reduction in the strength of naturally immunity, and potentially a paradoxical increase risk of infection in adults.

The data on naturally acquired immunity should also help identify why certain risk groups such as the elderly, patients with chronic lung disease, or HIV infection are highly susceptible to *S. pneumoniae* infections. Studies performed both in mice and patients have highlighted the phenomenon of immunosenescence (87, 88), including an increase in baseline inflammation that seems to impair the development of immunity against *S. pneumoniae* after colonization (88, 89). In the elderly the reduced frequency of colonization events (15, 90) and the decreased levels and activity of IgM memory B cells (91) could also have an impact on colonization-induced immunity to *S. pneumoniae*. *Streptococcus pneumoniae* specific CD4 responses are dysregulated by HIV infection (92), and these may underpin the greatly increased susceptibility of HIV positive subjects to *S. pneumoniae* infections. How age and comorbidity affects naturally acquired adaptive responses needs to be clearly defined so that there is a much clearer understanding why these groups are highly susceptible to *S. pneumoniae*. Effective methods of measuring the strength of antiprotein and CD4^+^ cellular immune response to *S. pneumoniae* could also considerable improve our ability to measure susceptibility to this pathogen, which at present depends on measuring anticapsular antibody levels alone.

The recognition that there is a significant element on natural adaptive immunity to *S. pneumoniae* should also provide opportunities for new strategies of vaccination for prevention of *S. pneumoniae* infections. Firstly, strategies could be developed that boost existing naturally acquired immunity, especially in high risk subjects. Secondly, by knowing the mechanisms required for preventing specific diseases such as pneumonia these could be targeted by novel vaccines, for example by aiming to induce CD4^+^ cellular (mainly Th17) mediated immunity. Furthermore, the recognition that protein antigens in humans can provide significant immunity should boost research into vaccines that induce immune responses to protein antigen.

## Summary

This review has discussed the induction and mechanisms of the naturally acquired immune response to *S. pneumoniae*. Multiple lines of evidence from animal experiments and in humans have convincingly demonstrated that nasopharyngeal colonization stimulates significant levels of naturally acquired immunity to *S. pneumoniae* that helps prevent subsequent colonization events as well as systemic and pulmonary infections. The mechanisms involved in preventing infection depends on anatomical site, with an emphasis on antibody during systemic infection and on Th17 CD4^+^ cells for mucosal infection, with pneumonia seemingly a combination of the two. In contrast to previous assumptions, antibody to protein as well as capsular antigens has an important role. Future work is required to delineate which protein antigens are the most important for protective immunity and whether there are important roles for other cellular immune mechanisms [e.g., Tregs, which have been shown to be important for innate immunity (93)] as well as Th17 CD4^+^ cells. Overall, these findings may lead to a much better understanding of why certain patient groups are at high risk of *S. pneumoniae* infection and should help improve future vaccine strategies.

## Author Contributions

ER-S, GE, and JB contributed to draft the manuscript. ER-S and JB contributed to revising it critically and wrote the final version.

### Conflict of Interest Statement

The authors declare that the research was conducted in the absence of any commercial or financial relationships that could be construed as a potential conflict of interest.
